# How does motion affect GABA-measurements? Order statistic filtering compared to conventional analysis of MEGA-PRESS MRS

**DOI:** 10.1371/journal.pone.0177795

**Published:** 2017-05-16

**Authors:** Sofie Tapper, Anders Tisell, Peter Lundberg

**Affiliations:** 1 Center for Medical Image Science and Visualization, Linköping University, Linköping, Sweden; 2 Radiation Physics, Department of Medical and Health Sciences, Linköping University, Linköping, Sweden; 3 Radiology, Department of Medical and Health Sciences, Linköping University, Linköping, Sweden; Indiana University, UNITED STATES

## Abstract

**Purpose:**

The aim of this study was to evaluate two post-processing techniques applied to MRS MEGA-PRESS data influenced by motion-induced artifacts. In contrast to the conventional averaging technique, order statistic filtering (OSF) is a known method for artifact reduction. Therefore, this method may be suitable to incorporate in the GABA quantification.

**Methods:**

Twelve healthy volunteers were scanned three times using a 3 T MR system. One measurement protocol consisted of two MEGA-PRESS measurements, one reference measurement and one measurement including head motions. The resulting datasets were analyzed with the standard averaging technique and with the OSF-technique in two schemes; filtering phase cycles ‘RAW PC’ and filtering dynamics ‘RAW Dyn’.

**Results:**

The datasets containing artifacts resulted in an underestimation of the concentrations. There was a trend for the OSF-technique to compensate for this reduction when quantifying SNR-intense signals. However, there was no indication that OSF improved the estimated GABA concentrations. Moreover, when only considering the reference measurements, the OSF technique was equally as effective as averaging, which suggests that the techniques are interchangeable.

**Conclusion:**

OSF performed equally well as the conventional averaging technique for low-SNR signals. For high-SNR signals, OSF performed better and thus could be considered for routine usage.

## Introduction

### Artifact reduction in MRS

When performing Magnetic Resonance Spectroscopy (MRS) measurements, it is necessary to achieve high spectral quality and to obtain reliable metabolite concentrations from the spectra with minimal effort. However, long acquisition times or certain clinical conditions (*e*.*g*., narcolepsy, Parkinson’s disease, and fibromyalgia) are circumstances that may lead to the formation of signal artifacts induced by subject movements during the MRS measurement (*e*.*g*. reduction in peak height or random signal spikes in the spectrum). Such artifacts influence the resulting spectrum negatively and quantification software may interpret and fit these artifacts as actual metabolite signals. These misinterpretations would most likely result in under- or overestimations of some or all metabolite concentrations investigated, which potentially could be disastrous in clinical research applications that sometimes depend on the detection of minute concentration differences. Moreover, it is not as simple to spot these artifacts in the MRS data compared to imaging data where the artifacts are more obvious in the resulting image. For this reason, it is important to have an algorithm that filters out the artifacts present in the data.

Several techniques for motion correction, both retrospective and prospective, have been evaluated previously in other studies focused on MRS applications. The two main prospective techniques evaluated for MRS data either use an MR navigator [1] or an optical system [2] for the real-time tracking of subject motion. Moreover, spectral registration [3] and ‘Order Statistic Filtering’ (OSF) using the median filter [4], are two retrospective techniques, both of which have been evaluated using time domain Point RESolved Spectroscopy (PRESS) data.

**Fig 1:**
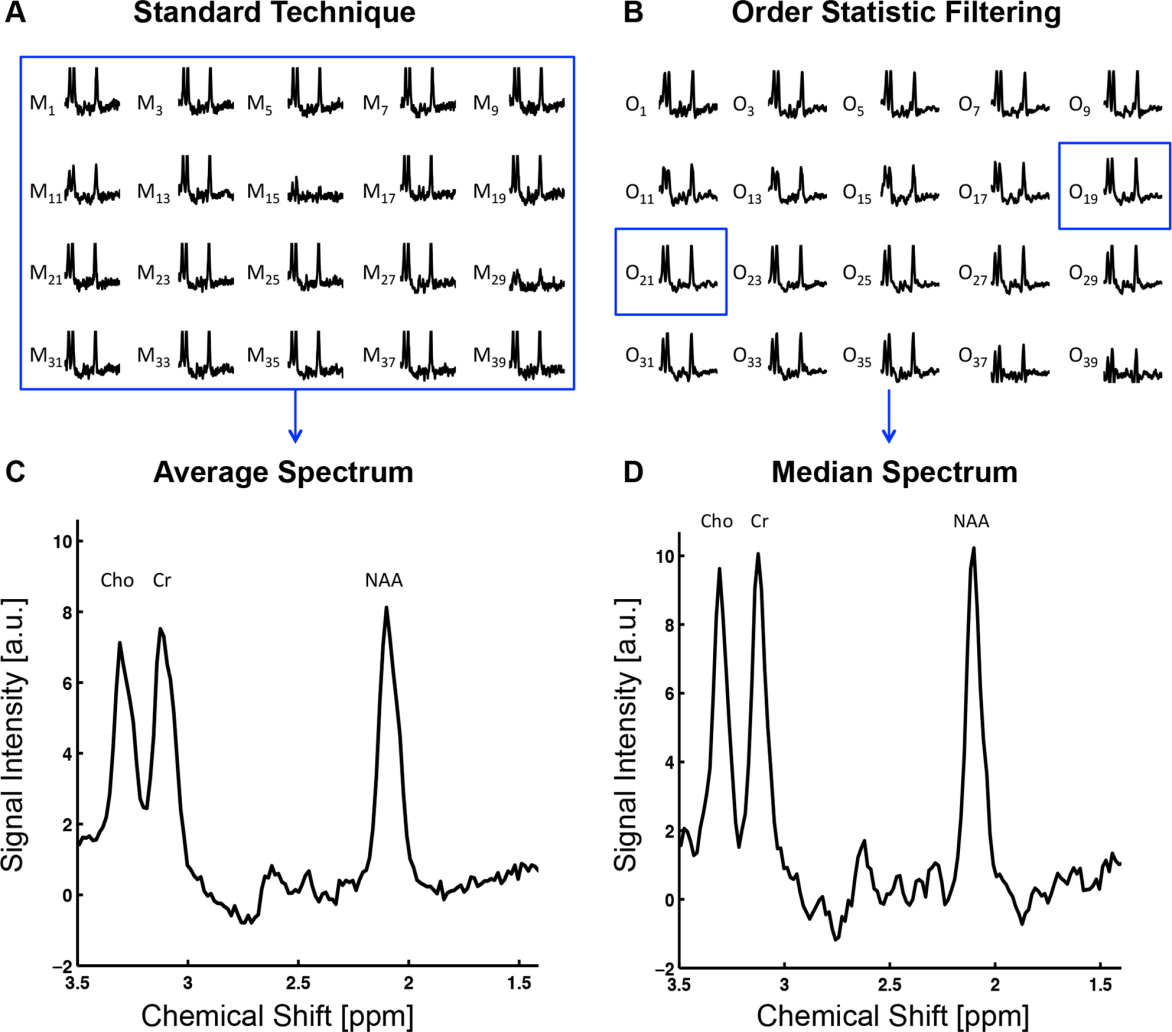
Comparison between the standard technique of averaging the spectra and OSF, computed for a typical measurement with induced artifacts (only showing OFF-spectra). (A) The standard approach of averaging all the acquisitions. The subject movement artifacts are clearly visible in the dynamics M_11_, M_15_ and M_29_. (B) The output from the OSF technique performed on the same dataset as in (A). The median spectrum is computed from the output (O_19_ and O_21_) and is used in the quantification. (C) The resulting averaged spectrum computed using the standard technique. (D) The resulting median spectrum using the OSF technique. It is clear that the signals in the median spectrum are more intense and that this technique provided a higher effective spectral resolution when artifacts influenced the data.

When performing edited MRS, a signal that is difficult to quantify is being targeted. For example, the signal may be obscured by another more intense signal in the spectrum. Moreover, subject movements and other disturbances, may change the phase of the signal, which results in a loss of signal. Edited MRS is therefore often highly susceptible to these small disturbances, and progress in both recognizing artifacts and improving the quality of motion-corrupted data is therefore clearly desirable. The OSF-technique has been shown to reduce motion artifacts in conventional MR-spectra of certain tissues and appears to also be an alternative for edited spectra of the brain [4]. The main disadvantage of the standard technique of simply averaging the sequence of spectra is that the artifacts are incorporated in the resulting spectra. In Fig 1, the standard and OSF-technique are compared side-by-side using a dataset containing artifacts induced by subject head movements. In the OSF-technique, each sample point in the spectral sequence is rank-ordered in the time-domain, *i*.*e*. sorted from most to least intense amplitude generating a new sequence of spectra, where the first and last spectrum contains the most intense and the least intense time-domain sample points, respectively. After rank-ordering, the median filter is applied to the time-domain data and the resulting median spectrum is quantified.

The OSF-technique has been shown to give more reliable spectroscopic data when artifacts induced by intentional subject motion influenced the data [4]. However, no evaluation of the OSF-technique in combination with metabolite quantification has been published previously.

### GABA quantification

γ-Amino-Butyric Acid (GABA) acts as the main inhibitory neurotransmitter in the mature human brain and is synthesized from glutamate, which acts as the main excitatory neurotransmitter in the mammal central nervous system [5, 6]. These two neurotransmitters coexist in the healthy human brain and several studies have shown a connection between regional altered GABA concentrations and neurological disorders [7–9]. Therefore, the implementation of a robust method for GABA quantification is essential to gain a better understanding of the human brain, and perhaps in the future, facilitating the incorporation of the GABA quantification in clinical diagnosis of neurological disorders. Moreover, one of the regions in the brain important in motion-disorders is cerebellum, and a focus of this work was therefore primarily on exploring the properties of data from cerebellum, and how they are affected by motion.

It is typically very challenging to perform the GABA quantification using MRS due to the low signal-to-noise-ratio (SNR) for the GABA signal relative to *e*.*g*. creatine, choline, NAA and water. Additionally, special spectral editing techniques are required to reveal the GABA-resonance at 3.01 ppm that is obscured by the much more intense creatine-resonance at 3.00 ppm. 'Mescher-Garwood Point RESolved Spectroscopy' (MEGA-PRESS) is currently the most commonly used pulse sequence for GABA detection [10–13]. This pulse sequence consists of a single-voxel PRESS sequence with two editing pulses applied alternately at 1.90 ppm and 7.46 ppm resulting in an alternating sequence of ON- and OFF-dynamics [10]. These ON- and OFF-dynamics are phase- and frequency corrected and then pairwise subtraction is performed to remove the creatine-signal, which results in a difference spectrum that reveals the GABA-resonance at 3.01 ppm [11]. The corresponding GABA-concentration is conventionally computed using a quantification program (*e*.*g*. LCModel [14]) with the average difference spectrum calculated from a predefined number of acquisitions used as input data. In this study, no macromolecular suppression [15, 16] was used in the MRS acquisitions, hence the usage of the term 'GABA+ concentrations' throughout this report (GABA+ is the sum of GABA and macromolecular background).

### RAW data

Raw data ('RAW') from an MRS-data acquisition contains the unprocessed data, thus including many more data points than the typically stored phase cycled data averages (*e*.*g*., SPAR/SDAT data on the Philips platform), which are normally available for spectral data processing. It is important to realize that these RAW-data therefore are *not* the conventional averaged 'raw'-data used for typical spectral processing, but data obtained prior to any intermediate processing and averaging applied by the scanner software. Thus, from such RAW data, it is possible to obtain the free induction decay from each *individual* phase cycle step and also *each separate coil element* for *each* ON- and OFF-dynamic. For example, one dynamic in the MEGA-PRESS dataset therefore usually consists of eight phase cycle steps that have been averaged, each taking the repetition time (TR) to acquire. Furthermore, if subject movement or signal artifacts only contaminate a single one of these phase cycle steps, the whole corresponding dynamic (complete phase cycle) becomes unreliable for quantification. Our hypothesis was that the OSF-technique would provide a better result if applied to the phase cycle steps available in the RAW data separately, than to the dynamics since the artifacts then can be removed prior to averaging the complete phase cycles.

We are not aware of any previously published work using RAW data obtained from MEGA-PRESS MRS acquisitions and especially not in combination with OSF. Moreover, only RAW data was used in this study since the scanner performs unknown post-processing steps (including procedures such as initial time-points intensity scaling) when the phase-cycled averages (here SPAR/SDAT files) are created from the RAW data.

### Aims

The primary aim of this study was to combine 'GABA+' quantification of the cerebellum with retrospective artifact reduction (OSF using the median filter) to explore the overall reliability of the computed GABA+ concentrations when intentional subject head motions influenced the data. Although our focus mainly is on motion disorders, the conclusions may be of general applicability. Another aim was to compare two main schemes, filtering phase cycles (‘RAW PC’), and filtering the dynamics (‘RAW Dyn’). The last aim in this project was to evaluate the performance of the OSF technique by investigating the concentrations obtained from LCModel for the more intense metabolite resonances glutamine and glutamate (Glx), N-acetylaspartate (NAA) and creatine obtained from solely analyzing the OFF-dynamics.

## Materials and methods

### Measurements

**Fig 2:**
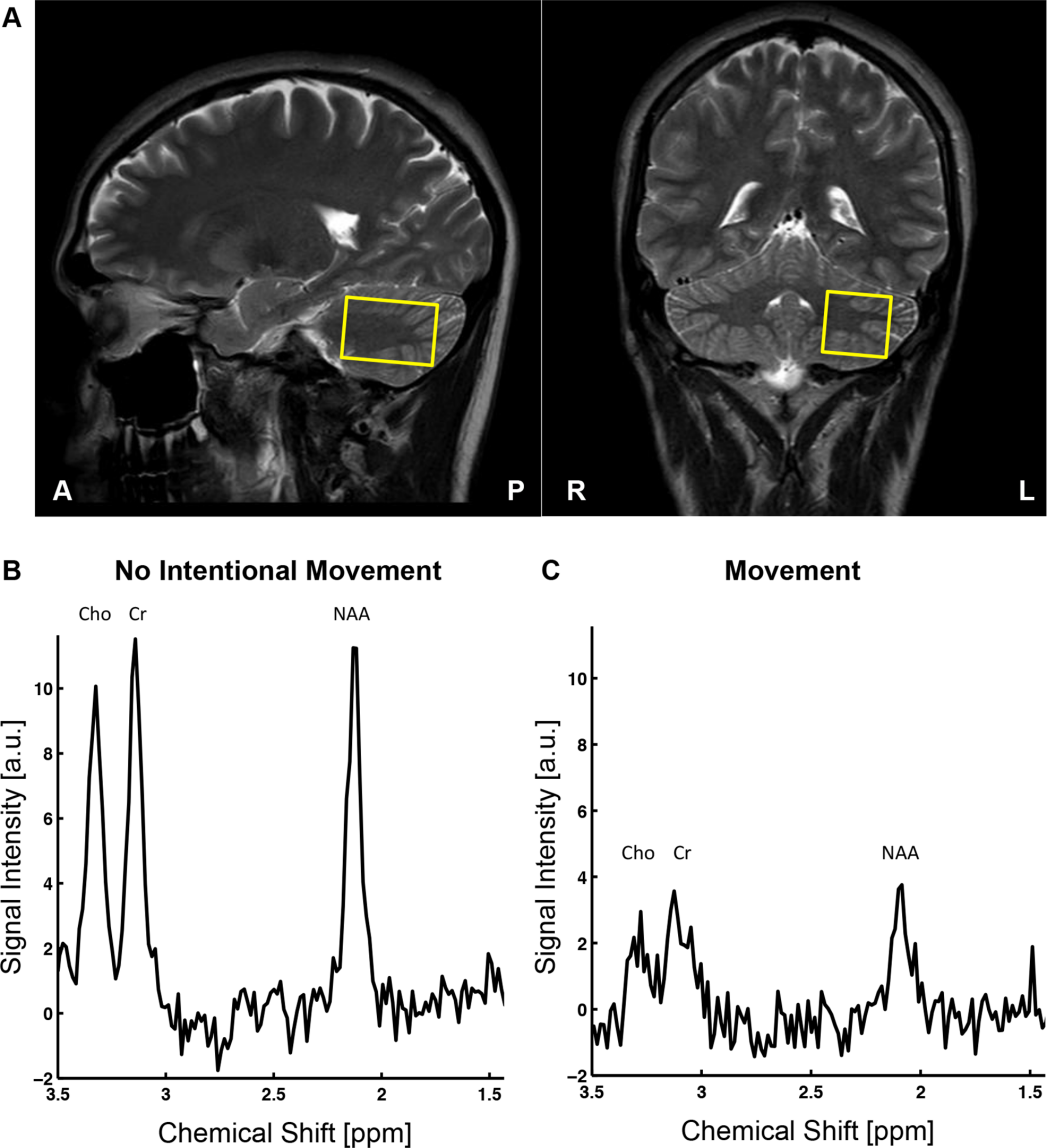
Typical voxel placement used during the acquisitions, and the effects on an OFF-dynamic due to motion-induced artifacts. (A) The voxel (3.5 x 2.5 x 2.5 cm^3^) was placed in the left region of the cerebellum in all the 12 healthy controls. (B) Spectrum computed from a single dynamic acquired from a measurement performed without any intentional movements. (C) Spectrum computed from a single dynamic with intentional subject movements performed during the acquisition. The movements alter the appearance of the spectrum by reducing the intensity of the resonances, where the amount of the reduction depends on the duration of the movements.

The Regional Ethical Review Board in Linköping approved this study (Dnr 2015 13–31), and written informed consent was obtained from all healthy volunteers. Twelve healthy volunteers, four males and eight females (22–59 years, mean age 29.5 years) were scanned using a Philips Ingenia 3 T MR system with a 12-channel phased array head coil. Moreover, an imaging sequence (T2 weighted, turbo spin echo sequence) was run to place the voxel (3.5 x 2.5 x 2.5 cm^3^, c. 22 mL) in the left region of the cerebellum according to Fig 2. The protocol consisted of two MEGA-PRESS measurements (320 transients, M = 40 dynamics, P = 8 phase cycles, N_raw_ = 16,384 samples (32 kHz) (*sic*), TR = 2 s, TE = 68 ms, editing pulses OFF at 7.46 ppm, editing pulses ON at 1.90 ppm, water suppression MOIST), the first measurement without any intentional movements (reference) and the second measurement containing four episodes of subject movement (randomized on which transient the movement began and a movement duration, between 1–8 transients, which corresponds to 2–16 s). The subject movement consisted of a head motion either in the up-down direction or in the left-right direction (approximately 20°). The subject could choose the movements that were most comfortable when in the scanner and the movements were conducted following commands given through the communication speakers available in the scanner. The subject was instructed to find the original placement of the head after each movement episode to have the voxel contained within approximately the same region throughout the whole protocol. Also, to verify that the voxel was still at approximately the same position, a short imaging sequence (T2 weighted, turbo spin echo sequence) was performed after each measurement (both the reference and the movement measurement). Moreover, the subjects were assumed to comply with the clear instructions provided, and thus the actual motion was not monitored using any additional hardware devices. Moreover, Fig 2 illustrates the effect of subject head movements on one OFF-dynamic compared to the same OFF-dynamic without any intentional movement.

To obtain a reference for water in the tissue, an identical but shorter unsuppressed water measurement (M = 2 dynamics, no water suppression) was collected before each MEGA-PRESS measurement. Furthermore, the protocol was performed three times for each volunteer (test-retest), which resulted in six distinct concentration estimates for every subject (and three of these estimates were influenced by motion-artifacts). Additionally, the RAW data was saved separately for all MRS measurements in each measurement protocol.

### Post-processing

**Fig 3:**
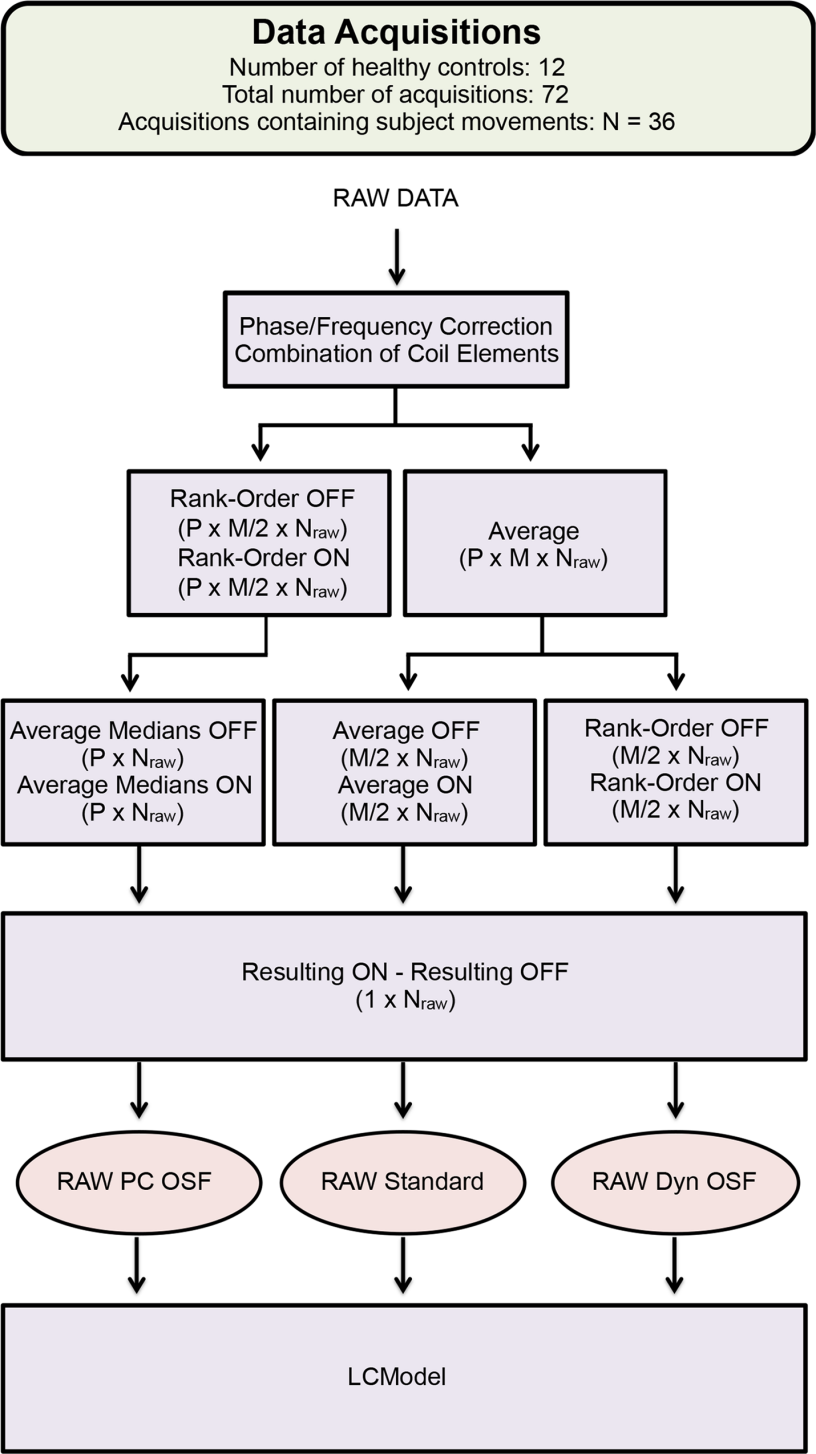
The workflow over the three different methods used for post-processing the MEGA-PRESS data obtained from the acquisitions, regardless of whether the measurement contained movements. The OSF was performed in two different ways using the RAW data, rank-ordering the phase cycles ‘RAW PC OSF’ and rank-ordering the dynamics ‘RAW Dyn OSF’, with the size of the data used shown in the parentheses.

After offline reconstruction using Reconframe (GyroTools, Switzerland), the RAW data had the dimension of P x M x N_raw_ x C, where C is the number of elements in the head coil. Each dataset, regardless of whether or not the dataset was influenced by subject motions, was post-processed in three different ways using the RAW data (Fig 3). First, the RAW data was phase-corrected according to Klose [17] and frequency-aligned based on the peak of the water residual. After correction, the coil elements were combined by weighting the SNR (Signal-to-Noise Ratio) computed from the water reference signal obtained from each coil element [18]. This first post-processing step generated the data (dimension of P x M x N_raw_) used to evaluate the three different schemes.

When applying the standard technique to the RAW data, the data was first averaged over the phase cycles and then over the dynamics, ON- and OFF-dynamics separately. Moreover, the OSF-technique was applied in two different ways, either rank-ordering the phase cycles ‘RAW PC’ or rank-ordering the dynamics ‘RAW Dyn’. After rank-ordering, the median ON- and OFF-dynamic was computed separately. Furthermore, for all three schemes, the resulting spectrum was computed by subtracting the resulting OFF-spectrum from the resulting ON-spectrum, which resulted in three resulting spectra (RAW PC OSF, RAW Standard and RAW Dyn OSF (Fig 3)) from each dataset. These three resulting difference spectra were quantified using LCModel (Version 6.3-1E) [14] with most current basis sets obtained from the laboratory of Dydak [19, 20], generating three separate estimates of the GABA+ concentrations for each data acquisition. The same procedure was performed one more time for solely the OFF-dynamics, generating three additional distinctive concentration estimations of total creatine, total N-acetyl compounds, and Glx for each measurement. All concentration estimates are available in the Supporting Information (S1 Data File).

### Statistical analyses

Correlation and Bland Altman plots [21] were used to illustrate how the resulting GABA+ and Creatine concentrations were distributed for each of the two techniques (standard and OSF). These plots were also used to investigate whether subject movements influenced the dataset or not. Moreover, paired t-tests were used to detect any significant differences in computed concentrations when considering the post-processing scheme used and whether the measurement contained motion-induced artifacts or not. Thus, paired t-tests were computed between the measurements containing motion-artifacts and the reference measurements for each scheme (Standard, PC, Dyn), and for each metabolite (GABA+, total creatine (tCr), total N-Acetyl compounds (tNA), Glx). Furthermore, to exclude the influence of the motion-artifacts, paired t-tests were also computed between the different schemes, for each metabolite, when only considering the reference measurements. To summarize, there were 24 paired t-tests performed in total, six for each metabolite, and of these six t-tests, three were detecting differences between the schemes and the other three were detecting differences due to motion-artifacts.

## Results

**Fig 4:**
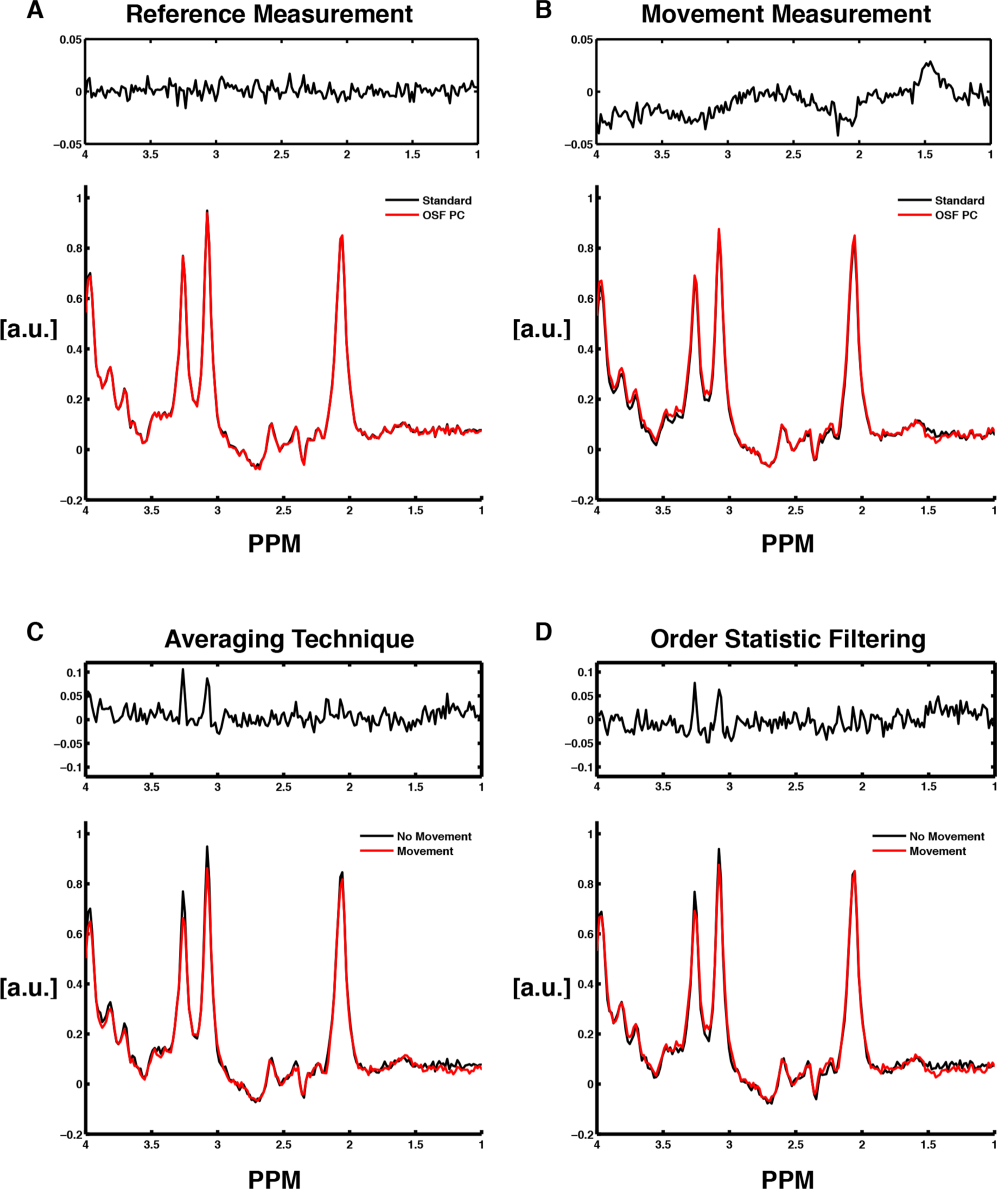
Resulting metabolite spectra obtained using the two different post-processing techniques applied to typical datasets from a measurement protocol (one reference measurement and one measurement containing motion-induced artifacts). The residuals between the two spectra are also showed over each pair of spectra. (A) Standard averaging and OSF (RAW PC) performed on a typical reference measurement. (B) Standard averaging and OSF (RAW PC) performed on a typical measurement containing motion-induced artifacts. (C) Standard averaging performed on a typical measurement with movements and on a typical reference measurement. (D) OSF (RAW PC) performed on a typical measurement with movements and on a typical reference measurement.

Fig 4 illustrates a typical case when comparing the resulting OFF spectrum from a reference measurement to a measurement with intentional movements, obtained from the same measurement protocol. The standard and OSF-technique performed equally well when no large artifacts were present. This was evident when looking at the residual between the spectra, showing that the residual was mostly white noise in the absence of artifacts. However, looking at the residual computed when the data contained motion-artifacts, the residual obtained a non-white noise appearance. Moreover, the signal intensity in the resulting spectrum was reduced when using the standard technique in the presence of these motion-induced artifacts. When evaluating the techniques separately, for the reference measurement and the following measurement with artifacts (Fig 4C and 4D), it was clear that both techniques lost signal intensity (choline and creatine) when the data contained movements. However, the residuals show that the NAA-signal was not as affected by this reduction in signal intensity as the other two singlets.

**Fig 5:**
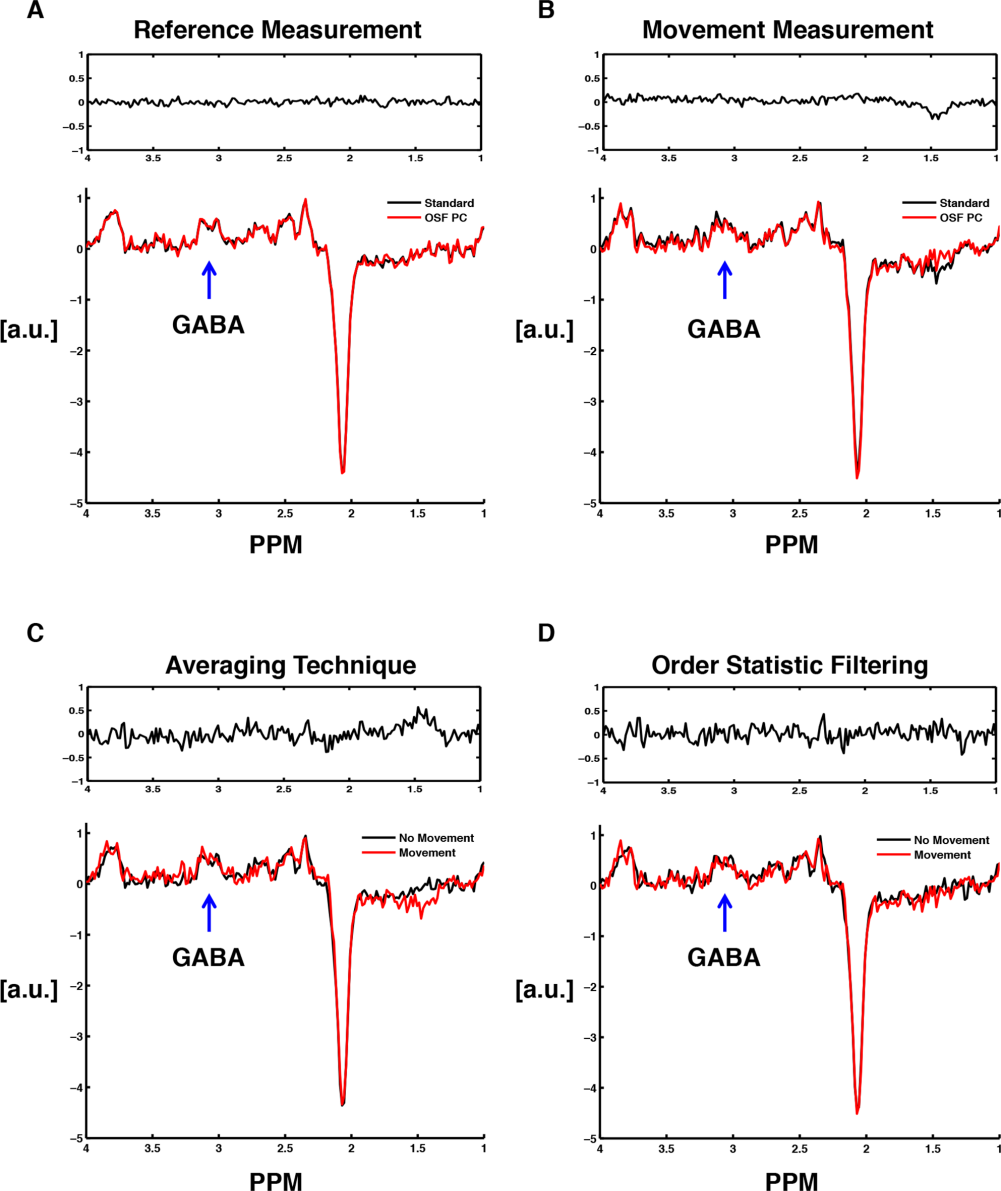
Resulting GABA difference spectra obtained using the two different post-processing techniques applied to the same datasets as in Fig 4. The residuals between each pair of spectra are also showed. (A) Standard averaging and OSF (RAW PC) performed on a typical reference measurement. (B) Standard averaging and OSF (RAW PC) performed on a typical measurement containing motion-induced artifacts. (C) Standard averaging performed on a typical measurement with movements and on a typical reference measurement. (D) OSF (RAW PC) performed on a typical measurement with movements and on a typical reference measurement.

Moreover, when inspecting the difference spectra (Fig 5) that resulted from the same datasets that were used in Fig 4, no obvious signal loss was observed. The residuals showed that there were no larger difference between the OSF and standard technique for GABA difference spectra. Thus, as seen by looking at the residuals in Fig 5, there were larger differences between the two datasets than the difference that resulted from using the two different post-processing methods.

### Standard technique vs. OSF technique

**Fig 6:**
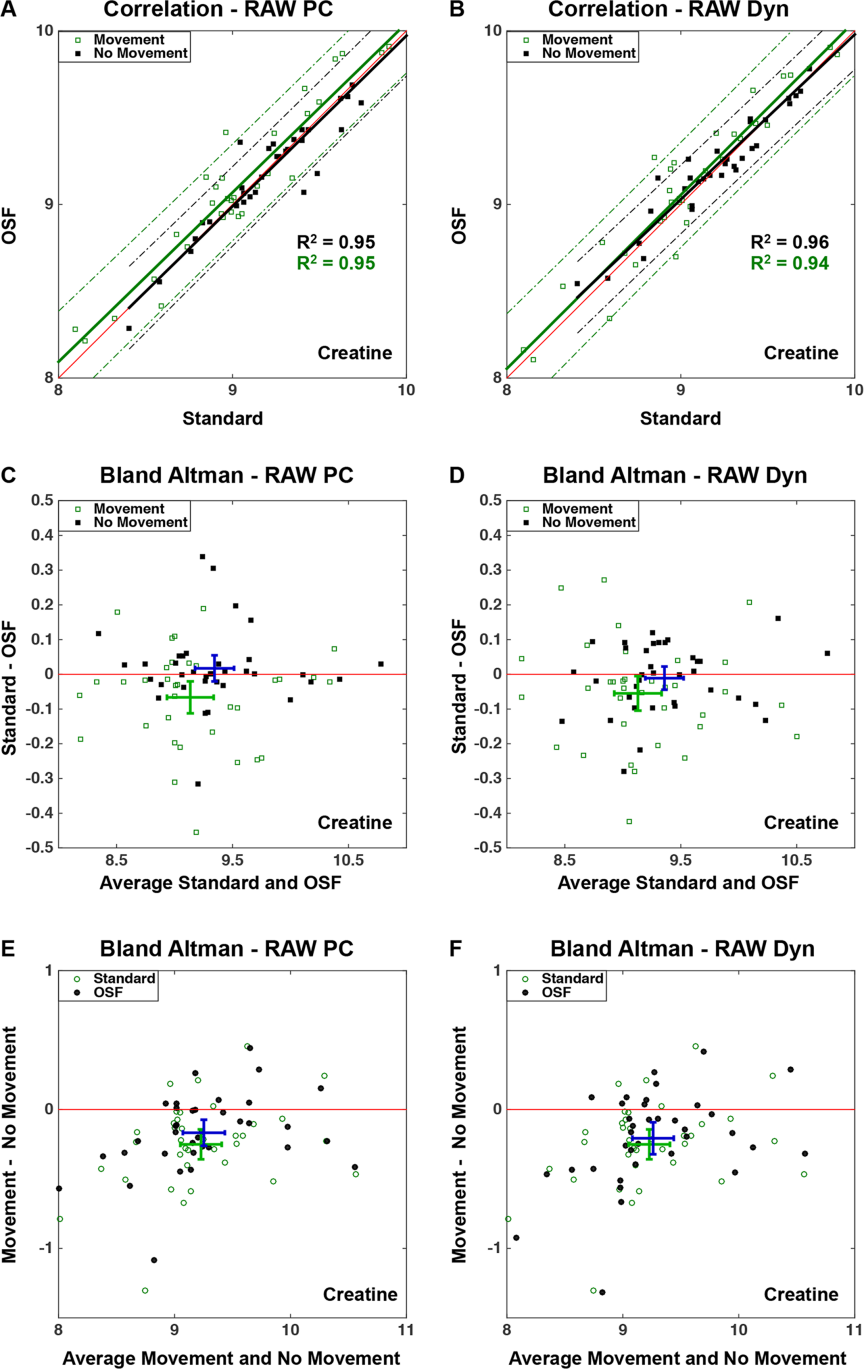
Correlation plots (A, B) and Bland Altman plots (C, D, E, F) comparing creatine concentrations computed using the three post-processing techniques described in Fig 3 applied solely to the OFF-dynamics, and comparing acquisitions with and without intentional movements. The correlation plots (A, B) show the linear relationship between the two OSF techniques and the standard technique, each having a linear fit with a 95% confidence interval plotted for the creatine concentrations with and without intentional movements. The Bland Altman plots (C, D) show the difference between the standard technique and OSF plotted against the average creatine concentration obtained from these two techniques, and (E, F) illustrates the differences in creatine concentrations between measurements with and without intentional movements. The mean difference and mean average are illustrated by the locations of the intersecting intervals for estimates without movements (C, D) and OSF (E, F) shown in dark blue, and with movements (C, D) and Standard technique (E, F) shown in green. The intersecting intervals illustrate the 95% confidence intervals of the mean difference and mean average.

**Fig 7:**
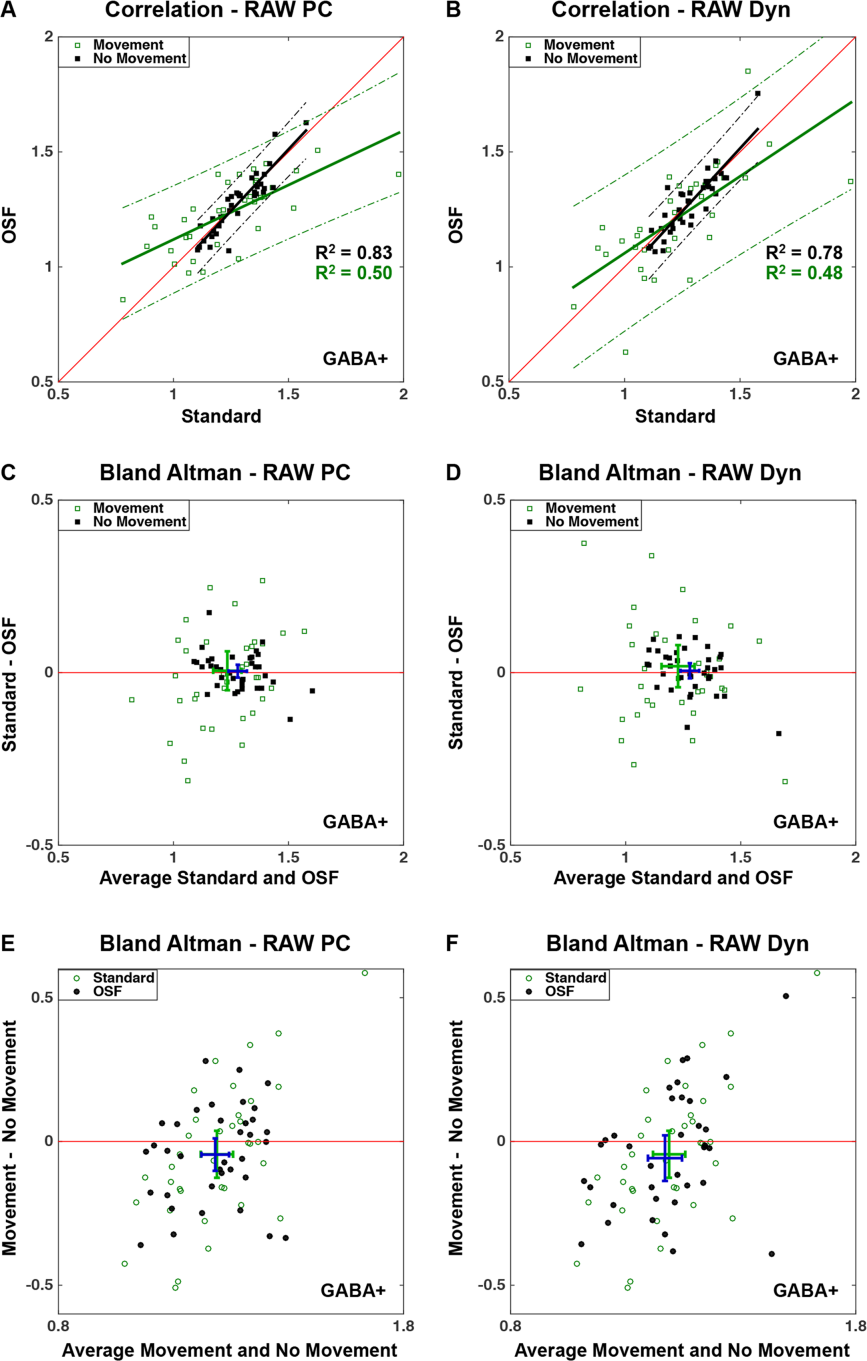
Correlation plots (A, B) and Bland Altman plots (C, D, E, F) comparing GABA+ concentrations computed for the three post-processing techniques described in Fig 3, and comparing acquisitions with and without movements. The correlation plots (A, B) show the linear relationship between the two OSF techniques and the standard technique, each having a linear fit with a 95% confidence interval plotted for the GABA+ concentrations with and without intentional movements. The Bland Altman plots (C, D) show the difference between the standard technique and OSF plotted against the average GABA+ concentration obtained from these two techniques, and (E, F) illustrates the differences in GABA+ concentrations between measurements with and without intentional movements. The mean difference and mean average are illustrated by the locations of the intersecting intervals for estimates without movements (C, D) and OSF (E, F) shown in dark blue, and with movements (C, D) and Standard technique (E, F) shown in green. The intersecting intervals illustrate the 95% confidence intervals of the mean difference and mean average.

A strong correlation was observed between the standard technique and the two OSF procedures for the acquisitions without any intentional movements (Figs 6A, 6B 7A and 7B). When the data contained motion-induced artifacts, the correlation between the techniques was still very high for creatine. Furthermore, Fig 6A and 6B shows a shift towards the OSF technique indicating that this technique increases the concentration estimates when the data contained motion-induced artifacts. However, the acquisitions with artifacts had a much weaker correlation between both techniques for GABA+ concentrations (Fig 7A and 7B). Moreover, the spread in concentrations was considerably larger for the measurements with artifacts compared to the reference measurements, which was obvious by observing the larger 95% confidence intervals for the measurements containing movements.

The Bland Altman plots (Figs 6C, 6D, 7C and 7D) confirm the previous observations; a higher concentration was observed when the OSF technique was applied to data that were affected by motion-artifacts, however, not for GABA+ concentrations where no larger differences were observed. When the measurements containing artifacts were analyzed, a larger spread in the resulting concentrations was observed, and more so for the GABA+ concentrations.

The motion artifacts thus reduced the metabolite concentrations, for both techniques and both schemes (Figs 6E, 6F, 7E and7F). This reduction was more evident for the standard technique, when observing creatine concentrations. However, this distinction between the standard and OSF technique was not observed for the GABA+ concentrations. Finally, there were not any larger differences observed between the two OSF schemes.

### Confidence intervals

**Fig 8:**
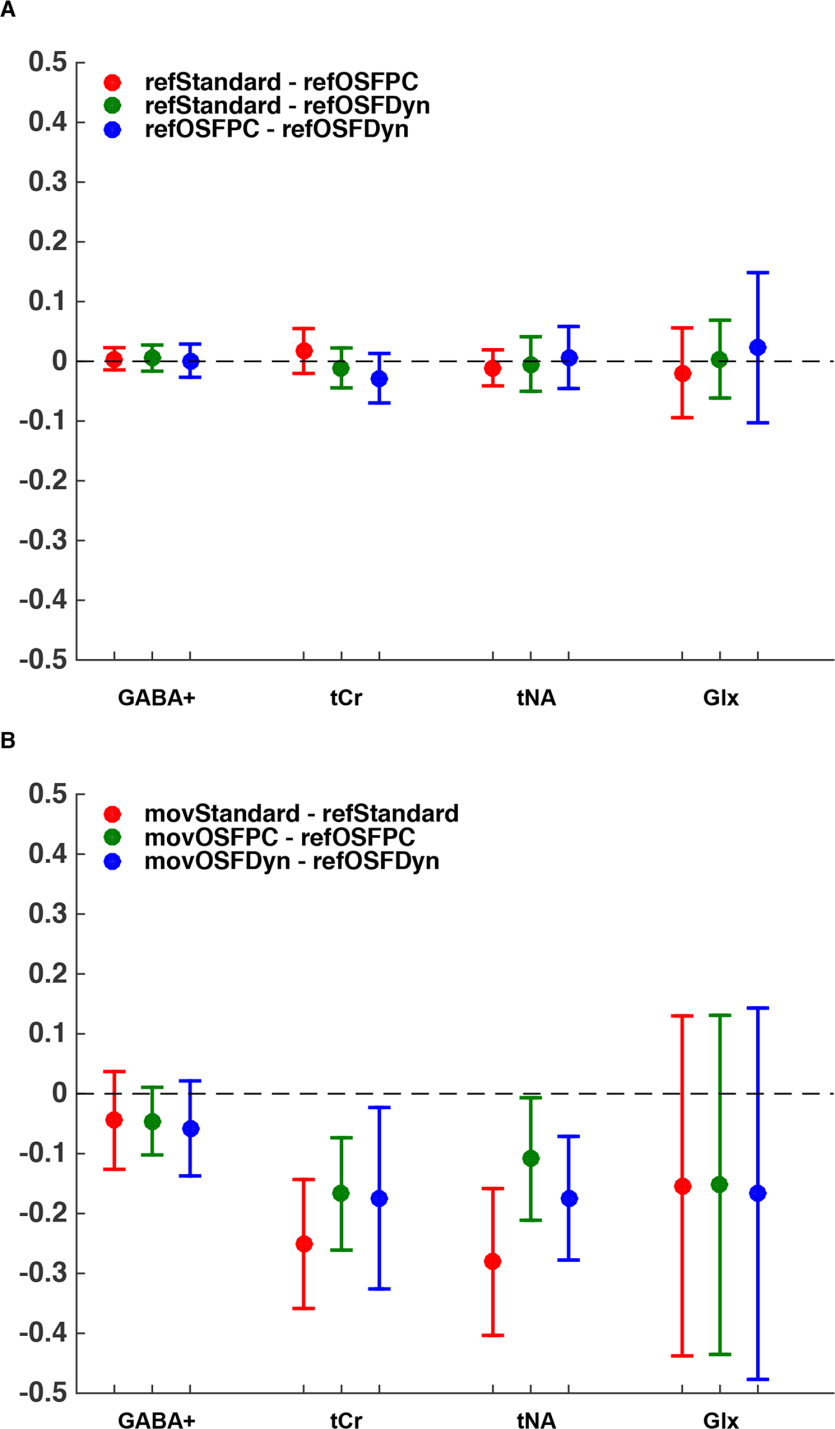
The confidence intervals computed from the paired t-tests performed for the GABA+, tCr, tNA and Glx concentrations. (A) Paired t-tests performed between the three different post-processing techniques applied only to the reference measurements. The three paired t-tests (refStandard–refOSFPC, red), (refStandard–refOSFDyn, green), and (refOSFPC–refOSFDyn, blue) were performed. (B) Paired t-tests performed between the measurements with movements and the reference measurements analyzed with the same post-processing method. The three paired t-tests (movStandard–refStandard, red), (movOSFPC–refOSFPC, green), and (movOSFDyn–refOSFDyn, blue) were performed.

Fig 8 shows the confidence intervals that resulted from the paired t-tests. The main observation was thus that the estimated concentrations were lower when motion-induced artifacts influenced the data, as indicated by the lower mean value of the confidence intervals. Moreover, there were no significant differences observed between the post-processing techniques when the measurements were performed without intentional movements (Fig 8A). When comparing the post-processing techniques for measurements with and without artifacts, the confidence intervals were much larger (Fig 8B). Furthermore, the more intense metabolites tCr and tNA, represented by spectral singlets, showed significant differences between the measurements with and without movements for all three post-processing schemes. An improvement was observed when using the OSF technique for the more intense metabolites tCr and tNA, however, still significantly lower than the reference measurement. Moreover, for GABA+ and Glx concentrations, there were no improvements observed using the OSF technique when artifacts were present. In addition, the confidence intervals were much larger for the Glx concentrations.

Moreover, when detecting differences between the reference measurement and the measurement containing artifacts (Fig 8B), the RAW PC scheme resulted in a slightly less difference in estimated concentrations than the RAW Dyn scheme. Also, smaller confidence intervals were observed for the RAW PC scheme.

## Discussion

### Standard vs. OSF-technique

When reference data with no induced artifacts was used, the OSF technique was at least equally as effective as the standard averaging technique. This result showed that the OSF-technique clearly could be used for all datasets, independent of whether artifacts were present in the data or not.

For MRS measurements affected by motion-induced artifacts, both OFF- and ON-spectra (in the GABA-sequence) had less intense resonances than the control experiments. This significant signal loss might be a result of loss of coherence during the measurement as a consequence of the subject movements performed during the application of the excitation pulses. Additionally, when inducing motion artifacts in the data, there is an effect on the main field homogeneity that also affects the shim of the voxel, which may be another reason for this observed signal loss. Moreover, this relative signal loss in combination with the averaging technique consequently resulted in reduced final concentrations computed by LCModel for all metabolites. Furthermore, since a clear spectral improvement was observed (Fig 4B) when the OSF-technique was applied on a dataset containing these artifacts, the expectation was to observe a similarly obvious improvement in the concentration estimates as well. This was not the case, and only a trend of improvement was observed for the most intense resonances, *i*.*e*., tCr and tNA, while no such improvement was observed for the 'low SNR' and scalar coupled metabolites GABA+ and Glx. Consequently, our results clearly showed that the OSF-technique was not as effective as we had anticipated, when motion-induced artifacts affected the data.

In contrast, a reduction in signal intensity was not detected for the singlet NAA-resonance. A possible explanation was in our view that the latter resonance has a shorter T2 than the comparatively sharp singlet resonances of Creatine and Choline, thus these resonances characterized by longer T2 appeared to be more strongly affected by a signal reduction.

There may be several explanations for the lack of improvement in the GABA+ concentrations computed using OSF. Firstly, the GABA+ quantification (a multiplet resonance) was entirely different from the quantification of the other metabolites, since twice the amount of data was used to compute the resulting difference spectrum (a combination of ON and OFF-edited spectra). Secondly, the GABA+ signal was characterized by a considerably lower SNR than the creatine and NAA singlets, which clearly affected the result from the OSF technique since the SNR for GABA+ may not be sufficiently high for the OSF to be useful for such a metabolite (*cf*. discussion in [4]). Moreover, no clear differences were observed between the post-processing techniques when examining the results from a typical measurement protocol (Fig 5). Therefore, since an underestimation of the GABA concentrations was evident in Fig 8, there might be a difference in how LCModel estimates the GABA concentrations depending whether the data contained movements or not.

With respect to Glx-detection, the echo time was adjusted to 68 ms in the acquisitions, as this is the standard echo time for GABA+ quantification, however it is much larger than the optimal TE for Glx quantification. Moreover, a likely additional explanation to why the Glx concentrations (from the combined multiplets of glutamate and glutamine) did not result in any significance was the relatively high uncertainty of the Glx concentrations estimates computed by LCModel.

### RAW PC vs. RAW Dyn

The initial hypothesis was that the RAW PC scheme was the most effective procedure due to the elimination of motion artifacts *prior to* the combination of the phase cycles. Therefore, the better performance of the RAW PC scheme, when observing tCr and tNA in Fig 8, could be a consequence of the expected earlier isolation of the artifacts. Thus, instead of one final median that is obtained using the RAW Dyn scheme, in the RAW PC scheme, P = 8 reliable medians were averaged to the resulting spectrum. This difference apparently resulted in smaller standard deviations in the estimated concentrations for the RAW PC scheme.

### Inducing artifacts

The most straightforward way to introduce realistic motion artifacts in cerebellum when collecting *in vivo* MRS data was to instruct the volunteer to perform controlled episodes of head movements. For the purpose of motion *correction* during MRS acquisitions, there are techniques [1, 2] that are suitable for such correction. However, motion correction was not the purpose of this report, and therefore, the issue with a slightly shifted voxel was for that reason not considered a disturbing problem. Mostly, since the cerebellum is a large brain region and a potential slight spatial shift of the voxel (c. 22 mL) would therefore not have any large impact on the resulting data. Also, a large single voxel in cerebellum is likely less affected by motion in comparison with a similarly large voxel in the frontal brain regions.

From our observation that the concentration estimates were larger, and the spread in concentrations was smaller, for the reference measurements (*i*.*e*., without motion), it was concluded that these measurements contained few macroscopic motion-related artifacts. Internal motion like swallowing, pulse or breathing were assumed to only have a limited influence on the results and were thus not considered to be a problem that would affect the interpretation of the results. Each volunteer was questioned after each protocol, and nobody expressed any difficulties of finding the approximate original position, or to understand when to move, thus these matters were also not considered as experimental problems.

One of the main advantages of the movement paradigm was that the volunteers could move their heads freely since the utilized analytical techniques were capable of managing the movements independently of the movement direction. Furthermore, the risk for unrealistic motions was considered minimal since there was not much room for large head movements within the tight head coil. In contrast, the main disadvantage with the movement paradigm was that it only resulted in a systematic reduction of the signal, which might be a possible explanation for the minimal improvement when using OSF in combination with the median filter.

### Limitations

There were a few limitations of the acquisitions and data analyses performed in this study, and the most influential issue was likely the impact of the chemical shift displacement error. This error is a large problem particularly when using a PRESS-family pulse sequence at high field strengths (≥ 3 T) and a limited available B1+ [22]. As a consequence of this effect, the voxel from which the water (residual) signal at 4.76 ppm originates does not spatially match the measured voxel for the metabolites estimated in this study, *e*.*g*. GABA+ signal at 3.01 ppm. Another closely related issue is the 4-compartment effect, which reduces the SNR of the GABA-signals [23, 24]. These problems were not addressed during the acquisitions in this paper. A possible solution to this problem is to replace the PRESS pulse sequence with a (semi-)LASER ('Localized by Adiabatic Selective Refocusing') pulse sequence [25] to minimize this error [1].

The noise in the resulting spectrum theoretically increases for median filtering compared to averaging [4], which is the only negative aspect of the OSF technique. This reduction in SNR might be compensated for either by using an MR system with higher magnetic field strength, or by decreasing the gradient heating during the measurement, which minimizes the frequency drift present during the acquisition (no intrinsic frequency stabilization was used in these experiments). However, in this study, we did not observe any larger frequency drift present during the measurements (< 3 Hz), and thus, the minor phase and frequency correction normally used was considered to be sufficient.

It is not only frequency drift present during the acquisition that has a negative impact on the GABA+ concentrations [26]; subtle subtraction artifacts are also a limitation. These artifacts are induced due to poor alignment in resonances between the ON and OFF spectra prior to the application of pairwise subtraction. One way of improving the alignment is to use spectral registration [3], which is a recent method for correction of phase and frequency that is not based on any specific resonance (*e*.*g*., the water or NAA-resonances). This property naturally leads to the future interesting experiment of combining the spectral registration with OSF (especially in selected cases where such instabilities may affect the data). Moreover, subtle effects from system and subject instabilities may also arise as a consequence of the presence of scalar couplings, such as those affecting GABA+ and Glx resonances, but not the singlets of Cho, Cr or NAA.

Whenever MRS is used, it would be highly useful to be able to describe the spectral quality possibly using characteristics, such as CRLB, FWHM, SNR, etc. LCModel, which was utilized to perform the required quantification, is the major software package that many researchers and clinicians normally use for metabolite quantification. It has also been shown to provide less biased metabolite concentrations than alternative packages (*e*.*g*. TARQUIN, jMRUI) in a recent spectral fitting challenge (ISMRM MRS workshop, Lake Constance 2016). Thus, LCModel is in our view a universally important part of the process for obtaining reliable concentrations. The assumption was therefore that LCModel handled every spectrum in the same manner, doing the best of the situation. Unfortunately, the quality related reported parameters obtained from the present 64-bit version of LCModel (Version 6.3-1L), *i*.*e*., the CRLB% (the relative Cramér-Rao Lower Bound, also known as 'SD%'), SNR and FWHM (Full Width at Half Maximum), only differed marginally between the techniques. For example, all estimated %SD for tCr and tNA were typically reported as being "2%", which indicated that the best model fit was obtained for the lowest concentrations (*i*.*e*., those that were affected by motion-induced artifacts!). Thus, the rounding-off to integer values of the lower bounds (such as '2 SD%') results in *de facto* ordinal values, which for that reason was not at all useful for interpreting our data in a meaningful manner.

Moreover, the reported FWHM estimates provided by the latest release of LCModel were even cruder than the %SD, which resulted in FWHM-values with relatively large increments. These properties of the FWHM and %SD complicated the possibility to perform a meaningful statistical analysis aiming for quality assurance based solely on these parameters [14]. Therefore, in our view there was no meaning in reporting the %SD and FWHM, instead the comparison to the reference measurement (which was done here) was considered to be the most appropriate quality control. The residual between the fit and the data can be obtained from LCModel (cf..COORD) and this residual might be useful for certain quality control procedures, although it was not used here.

Moreover, as described previously, there was an assumption that LCModel handled each dataset according to the same procedure. However, there might be different effects from the baseline estimations performed by LCModel. This theory may be an explanation for the lack of improvement using OSF for the estimation of GABA and Glx concentrations.

For the data in this paper, the particular individual that analyzed the data was not blinded to which of the datasets contained artifacts, but this might not always be the case in clinical practice. Slotboom et al. [4] suggested that before analyzing the MRS data, a so called κ-test, which is unbiased, should be performed to determine whether the data is reliable or not. Should it not pass this κ-test, the data should be post-processed with the OSF-technique; otherwise, the preference is to use the conventional technique of spectral averaging. This usage of the κ-test might be a good idea, however, this introduces difficulties in applications were it is desired that the post-processing is performed using an identical method for all datasets.

## Conclusions

The OSF technique is advantageous as it performed equally well or better than the standard technique, regardless of whether artifacts influenced the data or not. Importantly, when incorporating motion-induced artifacts in the data, all three post-processing methods underestimated the resulting metabolite concentrations. One might hypothesize that this is a result of lost coherence during the application of a pulse sequence in the presence of subject motion, which is something that would result in lower and thus negatively biased absolute concentrations. A trend of improvement was observed for the most intense singlet resonances creatine and N-acetylaspartate (no scalar couplings), when using the OSF technique on datasets affected by motion-induced artifacts. Thus, one could consider using this technique routinely for those metabolites.

Both the RAW PC and RAW Dyn scheme gave similar results, but the RAW PC scheme performed slightly better of the two. With respect to GABA+ measurements, no significant differences in concentrations were observed between the OSF- and the standard technique. Therefore our conclusion with respect to GABA+ is that it would be an important priority to improve SNR in the quantification, prior to the application of the procedures described here.

## Supporting information

S1 Data File This file contains the resulting GABA+, Creatine-, NAA-, and Glx-concentrations after using the post-processing methods in this study. After post-processing, the concentrations were computed by LCModel and summarized in this file prior to any statistical analyses. FPxx-y indicates subject xx and measurement y.
(XLSX)
